# The Influence of Acute and Chronic Exercise on Appetite and Appetite Regulation in Patients with Prediabetes or Type 2 Diabetes Mellitus—A Systematic Review

**DOI:** 10.3390/nu16081126

**Published:** 2024-04-11

**Authors:** Christoph Konitz, Leon Schwensfeier, Hans-Georg Predel, Christian Brinkmann

**Affiliations:** 1 Institute of Cardiovascular Research and Sport Medicine, German Sport University Cologne, 50933 Cologne, Germany; lschwensfeier@gmail.com (L.S.); predel@dshs-koeln.de (H.-G.P.); 2 Department of Fitness and Health, IST University of Applied Sciences, 40233 Düsseldorf, Germany

**Keywords:** exercise, appetite, satiety, diabetes, prediabetes

## Abstract

This systematic review aims to analyze the effects of acute and chronic exercise on appetite and appetite regulation in patients with abnormal glycemic control. PubMed, Web of Science, and the Cochrane Central Register of Controlled Trials were searched for eligible studies. The included studies had to report assessments of appetite (primary outcome). Levels of appetite-regulating hormones were analyzed as secondary outcomes (considered, if additionally reported). Seven studies with a total number of 211 patients with prediabetes or type 2 diabetes mellitus (T2DM) met the inclusion criteria. Ratings of hunger, satiety, fullness, prospective food consumption, nausea, and desire to eat, as well as levels of (des-)acylated ghrelin, glucagon-like peptide 1, glucose-dependent insulinotropic peptide, pancreatic polypeptide, peptide tyrosine tyrosine, leptin, and spexin were considered. Following acute exercise, the effects on appetite (measured up to one day post-exercise) varied, while there were either no changes or a decrease in appetite ratings following chronic exercise, both compared to control conditions (without exercise). These results were accompanied by inconsistent changes in appetite-regulating hormone levels. The overall risk of bias was low. The present results provide more evidence for an appetite-reducing rather than an appetite-increasing effect of (chronic) exercise on patients with prediabetes or T2DM. PROSPERO ID: CRD42023459322.

## 1. Introduction

Type 2 diabetes mellitus (T2DM) poses an enormous socio-economic burden, while the global prevalence of prediabetes and T2DM is steadily increasing. Between 1980 and 2021, the estimated number of people living with diabetes has risen from 108 million to 537 million, reflecting an increase in prevalence from 4.7% to 10.5% [1,2,3]. Overweight and obesity are strong risk factors for the development of diabetes [4], while weight reduction lowers diabetes risk [5]. Hence, maintaining or reaching a normal body weight is crucial for the prevention and treatment of diabetes.

Lifestyle interventions for weight loss and increased activity levels remain the preferred option for the prevention and treatment of T2DM due to their exceptional risk-to-reward and cost-to-benefit ratios [6,7]. Recent exercise recommendations for patients with prediabetes or T2DM are mainly based on studies that investigated the effects of exercise on health outcomes, such as glycemic control (HbA1c), insulin sensitivity, blood lipids, or blood pressure [7,8]. The impacts of exercise on appetite, energy balance, and consequently body weight are less studied in patients with prediabetes or T2DM, although it has been acknowledged for over 60 years that relevant interlinkages between these factors exist [9,10]. Therefore, a better understanding of how exercise affects appetite and appetite regulation in patients with prediabetes or T2DM may help improve current exercise recommendations.

Recent models of appetite regulation assume that a combination of tonic and episodic appetite signals is responsible for controlling energy intake at the physiological level [11].

Tonic signals largely depend on body composition and energy expenditure. An inverse correlation of fat mass and energy intake exists, which is severely weakened in obese individuals [12]. The most prominent molecule that tonically reduces energy intake is leptin, which is secreted in adipocytes [13]. However, the presence of increased leptin levels in obese individuals suggests that leptin’s inhibitory effects on energy intake are impaired in obese individuals [14]. This phenomenon is commonly referred to as “leptin resistance”.

Conversely, episodic signals play a role in the acute regulation of energy intake, for example, following food intake or exercise. The gastrointestinal peptides cholecystokinin (CCK) [15], glucagon-like peptide-1 (GLP-1) [16], pancreatic polypeptide (PP) [17], and peptide tyrosine tyrosine (PYY) [18] are notable anorexigenic hormones in this regard. These hormones are counteracted by the orexigenic peptide ghrelin [19]. Ghrelin exists in the two isoforms of des-acylated (dAG) and the biologically active form acylated ghrelin (AG) [20].

In the short term, exercise usually results in a relative energy deficit in individuals with normal glucose tolerance due to the additional calories expended during physical activity [21]. Immediately following acute exercise, a decrease in AG and increases in GLP-1, PP, and PYY suggest a reduction in appetite in individuals with normal glucose tolerance [22]. High-intensity exercise in particular has been shown to rapidly decrease hunger. This phenomenon is known as “exercise-induced anorexia” [23]. However, over longer periods of time, such an exercise-induced energy deficit is often diminished due to a compensatory increase in energy intake, as well as to a compensatory reduction in other areas of daily energy expenditure, e.g., non-exercise activity thermogenesis. Such compensatory responses demonstrate a high interindividual variation and seem to become more pronounced with higher exercise volumes [24,25,26].

Studies examining the long-term effects of chronic exercise on appetite regulation in healthy individuals are relatively rare. It is assumed that long-term exercise training increases fasting hunger but simultaneously improves acute satiety for a meal [27,28]. A chronic increase in hunger may be explained by a decrease in fat mass and a rise in fat-free mass [29,30].

The evidence of the influence of chronic exercise on episodic appetite signals is inconsistent. Some studies observed increases in AG, PYY, GLP-1, and PP [28,31,32,33], while others found no change in fasting or postprandial AG, PYY, or PP [34].

There are indications that appetite regulation is altered in patients with prediabetes or T2DM compared to individuals with normal glucose tolerance due to a dysregulation of central and peripheral sensitivity to appetite-regulating signals. Underlying mechanisms might be central insulin resistance [35], decreased sensitivity of the vagal nerve for chemical and mechanical appetite-mediating signals from the gastrointestinal tract, and impaired hippocampal functioning, which has been shown to play a role in the regulation of food intake [36,37]. This also raises the question of whether the appetite-regulating effects of exercise on patients with prediabetes or T2DM differ considerably from those on people with normal glucose tolerance. Furthermore, the question of whether exercise may have undesirable effects, e.g., increased appetite and excessive compensatory eating responses that could interfere with the goal of weight loss, remains open. The aim of this systematic review was therefore to assess the effects of acute and chronic exercise on appetite and appetite regulation in patients with prediabetes or T2DM.

## 2. Methods

This systematic review was conducted based on the PRISMA (Preferred Reporting Items for Systematic Reviews and Meta-Analyses) guidelines [38].

### 2.1. Eligibility Criteria

The eligibility criteria of the included studies were developed in line with the PICO framework [39].

Studies were included in the review if all participants in at least one intervention group were diagnosed with either prediabetes or T2DM according to the American Diabetes Association’s (Arlington, VA, USA) definition [40]. Participants of both sexes were included, while studies involving under 18 year olds were excluded.

The included studies had to incorporate a bout of acute physical activity or a physical training intervention. Studies without specific exercise/training interventions (i.e., those that only provided general recommendations on physical activity) were excluded. A combination with dietary advice was accepted.

Trials had to allow for at least one of the following comparisons: 1. Between exercise and non-exercise control groups; 2. Between groups that followed different exercise regimes; or 3. Between participants with and without prediabetes or T2DM following the same exercise regime.

The included studies had to report ratings of appetite sensations. Hormone levels relevant for the regulation of appetite were considered as further outcomes if they were reported in addition to appetite ratings in the same study. Studies that measured hormones only without reporting appetite ratings were excluded. The reason for this was to allow for a direct comparison of changes in appetite ratings with changes in appetite hormones. Full texts had to be available in either English or German and published in a peer-reviewed journal.

### 2.2. Information Sources

A comprehensive search strategy was developed after conducting manual orientation searches and screening previous systematic reviews in this field to identify suitable search terms. The detailed search strings are presented in Table 1. PubMed, Web of Science, and the Cochrane Central Register of Controlled Trials (including results from Embase, CT.gov, ICTRP, and CINAHL) were searched for eligible studies up to 28 August 2023. The search was not limited to a specific start date. Additionally, the references of the included studies were screened for additional eligible studies.

### 2.3. Selection Process

After the removal of duplicates, the titles and abstracts of each identified record were reviewed for eligibility. In a next step, the full texts of identified records were retrieved and reviewed for eligibility. Each step was performed independently by two researchers (C.K. and L.S.), and any disagreement was discussed with a third researcher (C.B.) until a consensus was reached on which studies to include. If reports measured but did not report relevant outcomes, the corresponding author(s) was/were contacted to obtain the missing data. Rayyan.ai software was used for the selection process [41].

### 2.4. Data Collection Process

One researcher (C.K.) extracted the data from the included studies. If statistical information on reported appetite ratings was incomplete, the corresponding author(s) of the study was/were contacted to obtain the missing information. If studies displayed appetite ratings in graphs only and if no response was received from the corresponding author(s), the numeric values were extracted using a plot digitizer software (DigitizeIt 2.5) [42].

### 2.5. Data Items (Outcomes)

Information on appetite ratings, levels of appetite hormones, as well as the occurrence of adverse events were extracted from each included study. Furthermore, the author(s), title, publication year, number, sex, age, and body mass index (BMI) of participants, details of the exercise and diet regime(s), study duration, information on medication intake, and the study protocol were extracted.

### 2.6. Risk of Bias Assessment

The Physiotherapy Evidence Database (PEDro) scale was used for the risk of bias assessment [43]. This scale has already been used in previous systematic reviews to assess study designs that are comparable with those assessed in this review [44,45]. It consists of 11 items (for external validity (item 1), internal validity (items 2–9), and statistical reporting (items 10–11)). The final PEDro score was obtained by summing up the ratings of all items except item 1 (each item: yes = 1 or no = 0), resulting in a total score between 0 and 10. As the blinding of instructors and participants in exercise intervention studies is difficult but possible by using sham interventions, the corresponding items 5–6 were rated accordingly. Scores of 3 or less were considered “poor”, scores of 4–5 were considered “fair”, those of 6–8 were deemed “good”, and scores of 9–10 were considered to be of “excellent” quality. Two researchers (C.K. and L.S.) independently applied the PEDro scale to the included studies. Any divergences were discussed with a third researcher (C.B.) until a consensus was reached.

### 2.7. Synthesis Methods

Given the heterogeneity of exercise interventions and study designs, as well as the relatively small number of included studies, a narrative synthesis of the results instead of a meta-analysis was performed.

## 3. Results

### 3.1. Study Selection

**Figure 1:**
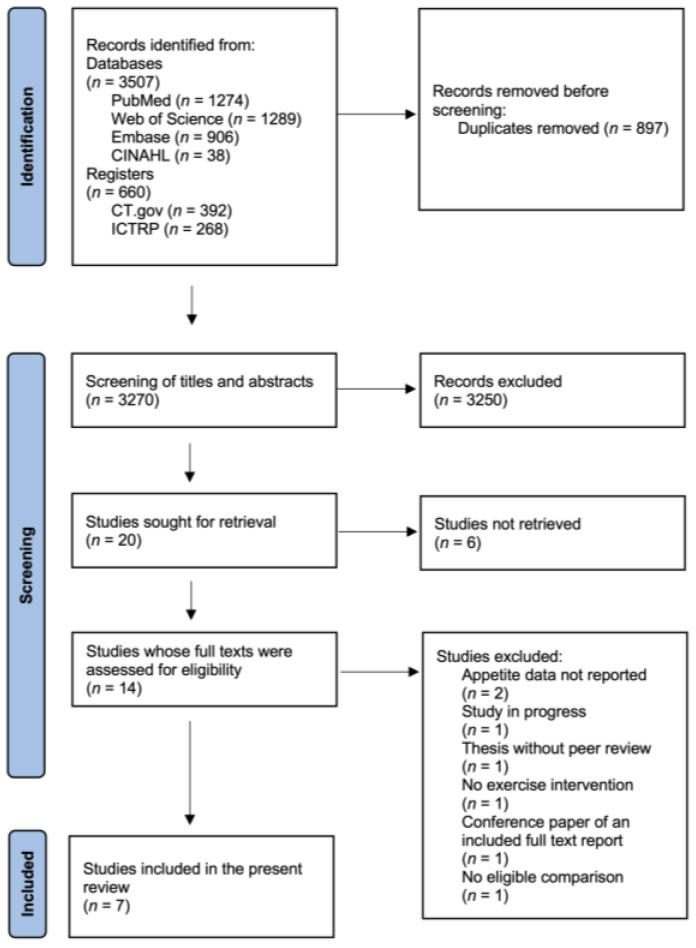
Prisma flow diagram.

Figure 1 shows the PRISMA flow diagram [38], which illustrates the study selection process. A total of 4167 records were identified from databases and registers. Seven studies met the inclusion criteria and were ultimately included.

### 3.2. Study Characteristics

**Figure 2:**
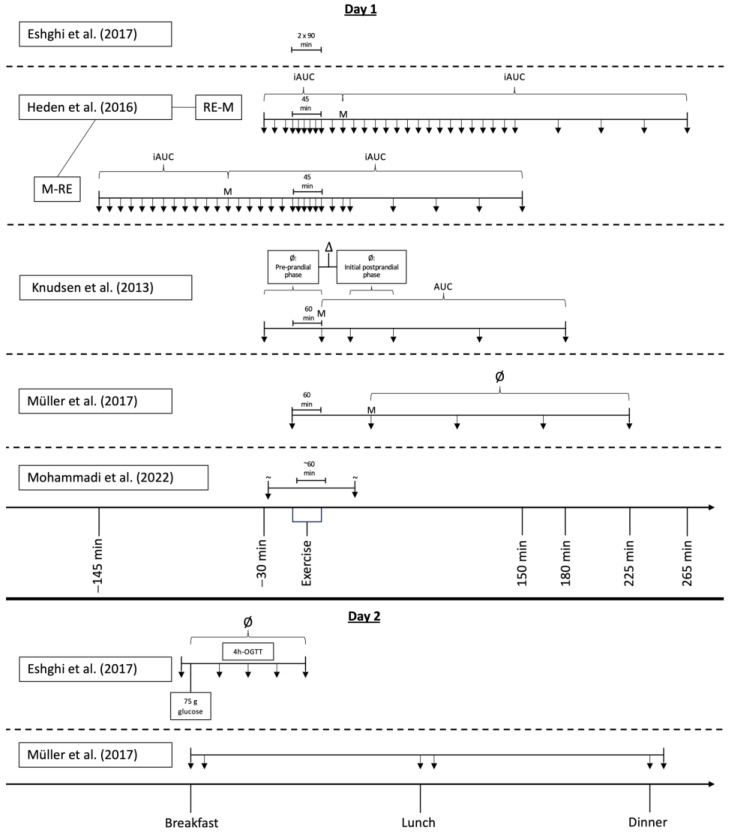
Timelines of studies investigating acute effects of exercise. (Ø, average; ∆, difference; (i)AUC, (incremental) area under the curve; OGTT, oral glucose tolerance test; M, meal or starting point of OGTT; ↓, measurement timepoint; ~, exact measurement timepoint not mentioned; solid line, dividing line between days; dashed lines, dividing lines between studies) [46,47,48,49,52].

The included studies were published between 2013 and 2023. Across all studies, a total of 211 participants (“study finishers”) were included. Twenty-eight participants were diagnosed with prediabetes and 183 participants with T2DM. Insulin-treated patients were not involved in any of the studies. Four studies investigated the effects of acute exercise on appetite [46,47,48,49], two studies the effects of chronic exercise [50,51], and one examined the effects of acute as well as chronic exercise [52] on appetite. The main characteristics of the included studies and study participants are presented in Table 2. More detailed information on the exercise and dietary regimes, as well as medication intake during the studies is available in Table 3. Figure 2 schematically presents the measurement timepoints and calculations of appetite ratings of studies investigating the acute effects of exercise.

### 3.3. Risk of Bias

Risk of bias assessments for each item are shown in Table 4.

Overall, the quality of evidence for both acute and chronic intervention effects can be rated as “good” according to the PEDro scale [43].

### 3.4. Effects of Acute Exercise on Appetite and Appetite Regulation in Patients with T2DM Compared to Control Conditions (without Exercise)

An overview of the results is presented in Table 5.

For assessments of hunger, satiety, prospective food consumption (PFC), and nausea, the directions of the effects within each acute study were relatively congruent, i.e., no single study, for example, showed a simultaneous increase in hunger and satiety. The individual rating scales within each study can therefore be translated into a general trend of appetite.

Following acute endurance exercise (aerobic or high-intensity endurance exercise), either no effect or the suppression of appetite was observed for up to 180 min after the training session [48,49]. One of two studies that measured appetite ratings the day after exercise [46,49] found an increase in appetite on day 2 [46].

Of the two studies that examined resistance exercise, one study identified appetite-suppressing effects at certain measurement timepoints [47], while the other study determined that resistance exercise acutely increased appetite [52]. In the latter study, the acute effect was re-examined after 12 weeks of training. The result remained unchanged and no chronic change in appetite ratings was observed.

The above-mentioned effects of endurance exercise on appetite ratings were inconsistently supported by changes in appetite hormones. Two studies observed no significant changes in the measured appetite hormones (ghrelin, PP, PYY, and leptin), although the feeling of fullness increased at certain measurement timepoints [48,49].

The increase in appetite the day after exercising that was observed in one study was not supported by corresponding changes in appetite hormones [49]. Instead, the small increases observed in GIP and GLP-1 would indicate a decrease in appetite. Of the two acute resistance exercise studies, only one examined the acute effects on appetite hormones [47]. The observed decrease in appetite at certain measurement timepoints was partially supported by a corresponding reduction in AG. A decrease in PP was not in line with the changes in appetite ratings.

### 3.5. Effects of Chronic Exercise on Appetite and Appetite Regulation in Patients with Prediabetes or T2DM

An overview of the results is provided in Table 6. The study by Heiston et al. [50] had two intervention groups but no control group, and was therefore not included in Table 6.

Either no changes or a decrease in appetite ratings was observed following chronic exercise. With a total number of 108 participants at baseline, the study by Vidanage et al. was the largest of the studies assessing chronic intervention effects [51]. Over the course of the study, hunger decreased and satiety increased in the aerobic endurance training group as well as in the group performing resistance training with resistance bands in addition to aerobic endurance exercise. Pre- and post-meal satiety increased in the combined training group, whereas in the endurance-only group, only pre-meal satiety increased after six months. It must be noted that the study’s protocol states that hunger, satiety, fullness, and PFC were measured, but only the results for hunger and satiety are reported. A request for the missing results forwarded to the study’s contact person did not yield a response.

In the study by Mohammadi et al., one group performed conventional resistance training, while the other engaged in aerobic endurance training [52]. No significant effects on satiety or fullness were observed in any intervention group throughout the study’s duration. The effects of acute exercise did not change during the intervention from pre- to post-training.

No significant effects on hunger were observed in any intervention group in a pre- to post-comparison in the study by Heiston et al. [50]. At 120 min during an oral glucose tolerance test (OGTT), fullness decreased in both intervention groups (continuous aerobic endurance training and HIIT) compared to pre-training. This study only lasted 2 weeks and was the shortest of the chronic intervention studies. It was the only one that involved patients with prediabetes and did not include a passive control group.

The above-mentioned chronic effects on appetite ratings were mostly not in line with changes in appetite hormones. In the study by Heiston et al., the postprandial increase in fullness did not correspond to the changes in the measured appetite hormones (ghrelin, GLP-1) [50]. Furthermore, changes in the fasting levels of GLP-1 developed in opposite directions in the AT and HIIT groups, although neither group showed a change in fasting appetite ratings.

Mohammadi et al. were the only researchers to examine the effects of exercise training on spexin [52]. Spexin is known to be an anorexigenic peptide in rodents and is thought to reduce energy intake in humans [55,56]. The chronic increase in fasting spexin concentration found from pre- to post-interventions in both groups was not associated with a change in appetite ratings.

### 3.6. Effects of Exercise on Appetite and Appetite Regulation in Patients with T2DM versus Normal Glucose Tolerance

Two acute studies allowed for a direct comparison between participants with and without T2DM [46,48].

In the study by Knudsen et al., no significant differences in appetite ratings were observed between the groups of subjects with and without T2DM when exercise was performed [48]. Without exercise (during the control trial), an OGTT decreased fullness in participants with T2DM while it increased fullness in those with NGT [48]. Moreover, the OGTT increased nausea to a significantly greater extent in participants with T2DM. The levels of total and acylated ghrelin were consistently lower in participants with T2DM, regardless of whether exercise was performed or not.

In the study by Eshghi et al., differences in fullness and desire to eat were observed between the groups of subjects with and without T2DM [46]. Postprandial fullness on the day after exercising only decreased in the group of subjects with T2DM [46]. Overall, the desire to eat something sweet was significantly lower in the T2DM group. However, exercise increased their desire to eat something sweet during an OGTT one day after exercising, while it decreased this craving in the group of subjects without T2DM.

### 3.7. Adverse Events and Patients’ Adherence to the Study Protocol

None of the included studies explicitly reported on whether adverse events occurred. There were two dropouts in the acute intervention studies. These were attributable to incomplete data collection [48] and personal reasons [49]. Most dropouts were observed in the chronic intervention studies. In the study by Heiston et al., four out of 32 participants dropped out for reasons unrelated to the intervention [50]. In the study by Mohammadi et al., eight out of 36 participants were excluded from the analysis [52]. The study’s authors did not provide any reasons for this exclusion. In the study by Vidanage et al., five out of 108 participants were excluded from the analysis due to poor dietary recording, poor dietary adherence, or loss of contact [51].

## 4. Discussion

The present review shows that the effects on appetite (measured up to one day post-exercise) following acute exercise vary in subjects with prediabetes or T2DM, while there are either no changes or a decrease in appetite ratings following chronic exercise compared to control conditions (resting conditions).

The most consistent finding from acute exercise studies is that the perception of fullness in patients with T2DM is increased immediately after exercise [47,48,49]. This suggests that exercise-induced anorexia may also be present in patients with abnormal glucose tolerance. As Eshghi et al. did not measure appetite immediately after the exercise sessions but only the day after, the measured decrease in fullness was not considered in the analysis of the immediate effect of exercise [46].

It has been suggested that a decrease in AG alongside increases in GLP-1, PP, and PYY in individuals with normal glucose tolerance levels mediate the effect of exercise-induced anorexia [57,58]. Accordingly, Heden et al. observed reduced hunger and an increased perception of fullness in patients with T2DM alongside reductions in AG following resistance exercise [47]. Interestingly, the study found no significant increases in PYY and PP. In the postprandial exercise trial, PP levels were even reduced during exercise. A decrease in PP levels alongside a reduction in appetite seems contradictory, as a drop in PP levels would be expected to increase appetite [17]. This illustrates that hormones should not be considered in isolation when assessing the effects on subjective appetite perception. In healthy individuals, both aerobic endurance and resistance exercise have been shown to acutely increase PP levels [59]. An impaired response to changes in PP levels in individuals with T2DM could serve as a possible explanation for the different results. The other included studies that examined ghrelin, PP, and PYY immediately after exercise found no significant effects on the levels of these hormones [48,49].

The only increase in appetite (ratings of hunger, PFC) immediately after exercise (resistance exercise) was observed in the study by Mohammadi et al. [52]. However, when compared to the control group, no significant effects on satiety and fullness were observed. Furthermore, the findings of Mohammadi et al. must be interpreted with caution, as this was the only acute study in which appetite assessments were carried out without relation to a test meal or an OGTT. Unfortunately, the study does not explicitly state whether the appetite assessment was carried out immediately after the training session or with a delay after exercise.

In the study by Eshghi et al. [46], in which energy intake was kept constant during the exercise and control trials, the increase in appetite (reduced fasting fullness, postprandial increase in prospective food consumption, and desire to eat something sweet during OGTT) observed on the day after the training session could be interpreted as a homeostatic response to the higher level of energy expended for the exercise on the previous day. However, there was no increase in hunger. Whether this would actually lead to an increased food/energy intake and consequently even to a positive energy balance is questionable.

Eshghi et al.’s study [46] was also the only study that found significant differences in post-exercise appetite ratings between participants with and without T2DM. Because the BMI did not differ between groups, it is likely that the observed differences were due to differences in diabetes status rather than body weight. 

Regarding the optimal choice of exercise, one study suggests that HIIT exercise is superior to moderate-intensity aerobic exercise for increasing fullness immediately post-exercise [49]. In general, endurance exercise might be preferable to resistance exercise to acutely reduce appetite, as two studies observed contradictory effects of resistance exercise [47,52]. Endurance exercise sessions that resulted in appetite-reducing effects were 60 min long with a minimum intensity of 50% of maximal work capacity (W_max_) [48,49].

In the long term, aerobic endurance exercise performed for at least 150 min per week (brisk walking, 3–5 times per week) or in combination with resistance exercise (20 min/day, 2–3 times per week) presented the best results by decreasing hunger and increasing satiety in participants with T2DM [51]. In another study, aerobic endurance exercise performed at slightly lower volumes (3 times per week, 10–35 min, 50–70% of maximal heart rate (HR_peak_)), and resistance exercise (3 times per week, 8 exercises, 3–4 sets/exercise, and 55–80% of 1-repetition maximum (1-RM)) failed to show a significant effect on appetite ratings [52].

An earlier systematic review based on a meta-analysis determined that chronic exercise training increased fasting appetite in overweight adults [60]. However, exercise training did not significantly increase total energy intake. How can this be explained? It has been assumed that exercise training can affect appetite regulation in two different ways [27]. Firstly, by increasing the feeling of hunger in the fasted state and, secondly, by increasing the satiety/fullness response to a meal, with the latter effect offsetting the first effect on total energy intake. This hypothesis is further supported by the fact that exercise training can, on average, enhance weight loss in overweight people, but these effects are usually small [61].

Our review does not confirm that chronic exercise increases fasting hunger in patients with prediabetes or T2DM compared to control conditions (without exercise), as the included studies only observed neutral effects on fasting hunger or an appetite-reducing effect [51,52]. However, our review rather confirms the second effect (increased satiety/fullness response to nutritional intake) in patients with prediabetes or T2DM [47,48,49,51]. This finding is promising. The lack of a rise in fasting hunger with a concomitant improvement in postprandial satiety suggests that the beneficial appetite-reducing effect of exercise and the possible consecutive reduction in energy intake might be even stronger among patients with abnormal glucose tolerance than among overweight individuals with a normal glucose tolerance level.

If optimally implemented, these effects could further increase the potential of exercise to slow down the progression of T2DM and its complications (e.g., diabetic retinopathy or neuropathy) [62,63].

Overall, the included studies do not allow for a clear association between ghrelin, PP, PYY, GIP, GLP-1, spexin, and the recorded changes in appetite ratings.

In one study, for example, acute increases in fullness were accompanied by a decline in acylated ghrelin [47]. In another study, an increase in GLP-1 and GIP with exercise on the following day did not translate into a suppression of appetite [46]. Chronic changes in spexin through exercise training did not appear to lead to chronic changes in the appetite perception of patients with T2DM [52].

### Limitations and Merits of the Present Review

This review specifically deals with appetite ratings. Previous literature has shown that exercise can have an impact on appetite and energy intake, but also on overall energy expenditure [64,65]. In addition, some research suggests that subjective ratings of appetite do not always correspond to actual energy intake [58]. Consequently, this review does not allow for conclusions on the question of whether the effects on appetite ultimately lead to changes in food intake and weight change in the study participants.

Another limitation of our review is the heterogeneity of the study protocols. This applies to both appetite assessments (which were carried out at different timepoints and in relation or not in relation to food intake or an OGTT) and exercise interventions (which differed in exercise type, frequency of exercise sessions, duration, or intensity). Furthermore, measurement timepoints and statistical methods varied considerably across studies. Variables, such as time of day, energy intake, or timing of exercise, and meal consumption, may also potentially influence appetite, further complicating comparisons across studies. Another factor is that the included studies report average values. It must be noted that there is an exceptionally high inter-individual variability in appetite responses to exercise [24].

Another point to consider is that the observed (anorectic) effects of exercise could have been influenced by the characteristics of the study’s target population, e.g., by their BMI. To directly investigate the influence of body weight on the effects of exercise on appetite and appetite regulation in addition to diabetes status, studies involving groups with a different body weight and composition would be required.

A further limitation of all included studies is that the interventions were not blinded to the participants. This may have resulted in unavoidable placebo effects, especially when the outcome is a subjective assessment of the participants. However, blinding in sports interventions is difficult, even with sham interventions.

One of the merits of this review is the specificity of the research questions. The characteristics of the participants were quite similar in all included studies. All included studies focused solely on the effects of exercise. No concomitant dietary or other lifestyle interventions were carried out, and diet was appropriately standardized between groups/trials in acute exercise studies and before testing in chronic exercise studies. Furthermore, the overall risk of bias of the included studies was low.

## 5. Conclusions

The present results provide more evidence for an appetite-reducing rather than an appetite-increasing effect of (chronic) exercise on patients with prediabetes or T2DM.

However, practitioners should consider possible inter-individual differences in appetite responses to exercise. Studies on the effects of training interventions on appetite that also investigate changes in free-living energy intake and components of energy expenditure are necessary to draw definite conclusions about the effectiveness of exercise in influencing long-term energy balance.

## Figures and Tables

**Table 1:** Search terms.

Databases	Search Terms
PubMed, Web of Science	(“diabetes” OR “diabetic” OR “T2D” OR “T2DM” OR “insulin resistance” OR “insulin-resistant” OR “prediabetes” OR “type 2 diabetes” OR “impaired glucose control”) AND (“physical activit*” OR “training” OR “exercise” OR “sport” OR “endurance activit*” OR “aerobic activit*” OR “resistance training” OR “strength training” OR “muscle-strengthening” OR “weight-lifting program” OR “high-intensity interval training” OR “HIIT” OR “physical conditioning” OR “walking” OR “sedentary time” OR “sedentary lifestyle” OR “sitting time”) AND (“energy intake” OR “caloric intake” OR “calorie intake” OR “food intake” OR “meal size” OR “energy density” OR “feeding behavio*” OR “food preference*” OR “eating behavio*” OR “satiation” OR “motivation to eat” OR “food choice” OR “food selection” OR “desire to eat” OR “food reward” OR “food craving*” OR “appetite” OR “hunger” OR “compensatory eating” OR “appetite control” OR “satiety” OR “fullness” OR “energy balance” OR “energy intake” OR “test meal” OR “prospective food consumption”) AND (“controlled” OR “control group” OR “trial”) All fields. No filters. No automatic term mapping.
Cochrane Central Register of Controlled Trials	(“diabetes” OR “diabetic” OR “T2D” OR “T2DM” OR “insulin resistance” OR “insulin-resistant” OR “prediabetes” OR “type 2 diabetes” OR “impaired glucose control”) AND (“physical activity” OR “training” OR “exercise” OR “sport” OR “endurance activity” OR “aerobic activity” OR “resistance training” OR “strength training” OR “muscle-strengthening” OR “weight-lifting program” OR “high-intensity interval training” OR “HIIT” OR “physical conditioning” OR “walking” OR “sedentary time” OR “sedentary lifestyle” OR “sitting time”) AND (“energy intake” OR “caloric intake” OR “calorie intake” OR “food intake” OR “meal size” OR “energy density” OR “feeding behavior” OR “food preference” OR “eating behavior” OR “satiation” OR “motivation to eat” OR “food choice” OR “food selection” OR “desire to eat” OR “food reward” OR “food craving” OR “appetite” OR “hunger” OR “compensatory eating” OR “appetite control” OR “satiety” OR “fullness” OR “energy balance” OR “energy intake” OR “test meal” OR “prospective food consumption”) AND (“controlled” OR “control group” OR “trial”) All fields. Filters: Trials. No searching of word variations.

**Table 2:** Characteristics of included studies.

Study	Study Design	Trials	Duration of Intervention	Participants’ Characteristics			Exercise Type	Appetite Ratings	Appetite Hormones
				*N* (m/f)	Age (Years)	BMI (kg/m^2^)			
Eshghi et al. (2017) [46]	Randomized crossover (2 groups)	- T2DM: AE - T2DM: Rest - NGT: AE - NGT: Rest	1 day	12 (12/0) T2DM: 6 NGT: 6	T2DM: 61 ± 9 NGT: 43 ± 11	T2DM: 25 ± 4 NGT: 27 ± 3	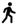	Hunger Fullness PFC Desire to eat something sweet, salty, or fatty	Total GIP GLP-1
Heden et al. (2016) [47]	Randomized crossover	- T2DM: Pre-meal RE (RE-M) - T2DM: Post-meal RE (M-RE) - T2DM: Rest	1 day	12 (5/7)	47 ± 12	37 ± 6	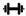	Hunger Fullness	AG PP PYY
Knudsen et al. (2013) [48]	Randomized crossover (2 groups)	- T2DM: AE - T2DM: Rest - NGT: AE - NGT: Rest	1 day	15 (15/0) T2DM: 8 NGT: 7	T2DM: 61 ± 2 NGT: 57 ± 1	T2DM: 28 ± 1 NGT: 27 ± 2	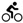	Hunger Fullness PFC Nausea	TG AG
Müller et al. (2017) [49]	Randomized crossover	- T2DM: AE - T2DM: HIIT - T2DM: Rest	1 day	13 (8/5)	65 ± 2	33 ± 1	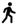	Hunger Fullness PFC Nausea	AG Leptin PP PYY
Mohammadi et al. (2022) [52]	Randomized parallel group design → PP	- T2DM: AE/AT - T2DM: RE/RT - T2DM: Rest (Control)	12 weeks	28 (28/0) AE/AT: 10 RE/RT: 9 Control: 9	≤50	AE/AT: 29 ± 2 RE/RT: 29 ± 2 Control: 29 ± 2	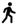 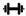	Hunger Satiety Fullness PFC	Spexin
Heiston et al. (2019) [50]	Randomized parallel group design → PP	- PreD: AT - PreD: HIIT	2 weeks	28 (6/22) AT: 14 (3/11) HIIT: 14 (3/11)	AT: 62 ± 2 HIIT: 60 ± 2	AT: 35 ± 2 HIIT: 32 ± 1	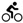	Hunger Fullness	AG dAG GLP-1
Vidanage et al. (2023) [51]	Randomized parallel group design → PP	- T2DM: AT - T2DM: AT + RT - T2DM: Rest (Control)	6 months	103 AT: 36 AT + RT: 34 Control: 33	AT: 49 ± 6 AT + RT: 53 ± 5 Control: 51 ± 5 (data from *n* = 36 in each group at baseline)	AT: 24 ± 4 AT + RT: 25 ± 4 Control: 25 ± 3 (data from *n* = 36 in each group at baseline)	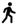 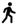 + 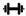	Hunger Satiety	-

AE/AT, aerobic endurance exercise/training; HIIT, high-intensity interval exercise/training; → PP, per protocol; RE/RT, resistance exercise/training; T2DM, type 2 diabetes mellitus; NGT, normal glucose tolerance; PFC, prospective food consumption; GIP, glucose-dependent insulinotropic peptide; GLP-1, glucagon-like peptide 1; (d)AG, (des-) acylated ghrelin; TG, total ghrelin; PP, pancreatic polypeptide; PYY, peptide YY; 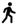, walking/running; 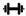, resistance exercise; 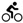, cycling.

**Table 3:** Detailed information on included studies.

Study	Exercise			Diet	Medication
	Type	Modality	Supervised		
Eshghi et al. (2017) [46]	Aerobic endurance exercise	Walking on a treadmill; 1-month exercise habituation phase before the intervention days (3 sessions per week at 80% of VT, until participants could walk for 90 min continuously); Two separate exercise bouts on one intervention day; 3.5 h rest between the two exercise bouts; 90 min per exercise bout; 80% of VT	Yes	Standardized diet with maintenance calories; two meals each after exercise on testing days. The first exercise bout was performed in the fasted state.	Metformin was paused 12 h before and during each testing day. No insulin.
Heden et al. (2016) [47]	Resistance exercise	Leg presses, seated calf raises, seated chest flies, seated back flies, back extensions, shoulder raises, leg curls, and abdominal crunches; 1 warm-up set, 2 working sets per exercise; 10 repetitions per set; 50% of 10-RM during warm up sets, 100% of 10-RM during working sets; 1–2 min rest between sets	Yes	Standardized diet with maintenance calories; three meals on testing days.	Usual medication (not specified) continued. No insulin.
Knudsen et al. (2013) [48]	Aerobic endurance exercise	Cycle ergometry; 60 min; 50% of W_max_, 60–90 rpm	Yes	Same mean energy intake and macronutrient distribution for 3 days on each trial. Controlled by using diet records. Exercise started after an overnight fasting period.	Antidiabetic, antihypertensive, and statin drugs were paused 3 days before and during each testing day. Drugs used: metformin (*n* = 7), DPP4 inhibitors and sulfonylureas (*n* = 1), antihypertensive drugs (*n* = 2), and statins (*n* = 4). No insulin.
Müller et al. (2017) [49]	Endurance exercise (aerobic (AE) and high-intensity exercise (HIIT))	Walking on a treadmill; 60 min; AE: 73% of VO_2peak_ HIIT: 3 min at 54% of VO_2peak_ followed by 3 min at 89% of VO_2peak_	Yes	Same diet for 24 h before intervention days. Achieved by replicating a diet record of the habitual diet of the participants during the first trial. Same standardized breakfast prior to exercise on the intervention days.	Antidiabetic medication (not specified) paused 2 days before and during each testing day. No insulin.
Mohammadi et al. (2022) [52]	Aerobic endurance training (AT) Resistance training (RT)	12 weeks; 3 sessions/week; AT: Walking/running on a treadmill; exercise duration and intensity increased over the course of the study; 10–15 min warm up, 10–35 min workout, 10–15 min cool down; 50–70% of HR_max_ RT: Leg presses, chest presses, leg extensions, leg flexions, lat pulldowns, overhead presses, arm curls, and triceps pushdowns; number of sets and intensity of 1-RM increased over the course of the study; 3–4 sets/exercise, 55–80% of 1-RM; 60–120 s rest between sets	Yes	Instruction to maintain habitual diet. Participants were instructed to consume the same diet during the week prior to the intervention and the week prior to the post-test evaluation. Controlled by using food frequency questionnaires.	Not further specified. Intake of any medication was forbidden 24 h before testing. No insulin.
Heiston et al. (2019) [50]	Endurance training (aerobic or HIIT)	12 sessions over 13 days; cycle ergometry; 60 min/session; AT: 70% of HR_peak_ HIIT: 3 min at 90% of HR_peak_ followed by 3 min of 50% of H_Rpeak_	Yes	Instruction to maintain habitual diet. Participants were instructed to consume a diet containing ~250 g of carbohydrates during the 24 h before pre-intervention testing. The diet record was replicated during the 24 h before post-intervention testing.	No antidiabetic or weight-loss-inducing medication. No insulin.
Vidanage et al. (2023) [51]	Aerobic endurance training (AT) Aerobic endurance training (AT) + resistance training (RT)	6 months; AT: Brisk walking; at least 150 min/week, 3–5 days/week; RT: Exercises with resistance bands for biceps, triceps, hamstrings, quadriceps, and pectoralis major muscle; 20 min/day; 2–3 days/week	No	No instructions.	Individuals on medication known to influence taste were excluded [53,54]. No insulin.

RM, repetition maximum; W_max_, maximal work capacity; VO_2peak_, maximal oxygen uptake; HR_peak_, maximal heart rate; VT, ventilatory threshold.

**Table 4:** Risk of bias assessment.

	Item 1 ^#^	Item 2	Item 3	Item 4	Item 5	Item 6	Item 7	Item 8	Item 9	Item 10	Item 11	Score
Eshghi et al. (2017) [46]	1	1	1	1	0	0	0	1	1	1	1	7
Heden et al. (2016) [47]	1	1	1	1	0	0	0	1	1	1	1	7
Knudsen et al. (2013) [48]	1	1	1	1	0	0	0	1	1	1	1	7
Müller et al. (2017) [49]	1	1	1	1	0	0	0	1	1	1	1	7
Mohammadi et al. (2022) [52]	1	1	1	1	0	0	1	0	0	1	1	6
Heiston et al. (2019) [50]	1	1	1	1	0	0	0	1	1	1	1	7
Vidanage et al. (2023) [51]	1	1	1	1	0	0	0	1	1	1	1	7

Item 1 = Eligibility criteria; Item 2 = Randomization; Item 3 = Concealed allocation; Item 4 = Similar prognostic indicators at baseline; Item 5 = Subjects blinded; Item 6 = Therapists blinded; Item 7 = Assessors blinded; Item 8: Retention rate of 85% or above; Item 9: Completeness of measurement results; Item 10 = Comparison between groups; Item 11 = Point measure and measures of variability. Note: ^#^ Item 1 did not contribute to the final score.

**Table 5:** Effects of acute exercise compared to control conditions (without exercise) in patients with type 2 diabetes mellitus (T2DM).

Study	Trial	Hunger	Fullness	PFC	Satiety	Nausea	Desire to Eat Sweet	AG	TG	PP	PYY	Leptin	GIP	GLP-1
Eshghi et al. (2017) [46]	AE	→	(→) ↓: In the fasted state on day 2	(→) ↑: Postprandial average during the 4 h OGTT on day 2	-	-	(→) ↑: Postprandial average during the 4 h OGTT on day 2	-	-	-	-	-	(→) ↑: After the 2nd exercise session on day 1 and in the fasted state on day 2	(→) ↑: In the fasted state on day 2
Heden et al. (2016) [47]	RE-M	(→) ↓: 25, 240, and 270 min post-exercise	(→) ↑: Pre-meal iAUC, during exercise (30 and 40 min after start), and 5 and 25 min after end of exercise	-	-	-	-	(→) ↓: Pre-meal iAUC, during exercise (40 min after start), and 25 and 60 min post-exercise	-	→	→	-	-	-
	M-RE	(→) ↓: 10, 120, and 150 min post-exercise	(→) ↑: 120 and 150 min post-exercise	-	-	-	-	→	-	(→) ↓: During exercise (15 min after start)	→	-	-	-
Knudsen et al. (2013) [48]	AE	→	(→) ↑: 180 min AUC post-exercise	→	-	→	-	→	→	-	-	-	-	-
Müller et al. (2017) [49]	HIIT	→	(→) ↑: 45 min post-exercise	→	-	→	-	→	-	→	→	→	-	-
	AE	→	→	→	-	→	-	→	-	→	→	→	-	-
Mohammadi et al. (2022) [52]	RE	↑ After the first and last exercise sessions ^1^	→	↑ After the first and last exercise sessions ^1^	→	-	-	-	-	-	-	-	-	-
	AE	→	→	→	→	-	-	-	-	-	-	-	-	-

AE, aerobic endurance exercise; RE-M, pre-prandial resistance exercise; M-RE, postprandial resistance exercise; HIIT, high-intensity interval exercise; RE, resistance exercise; PFC, prospective food consumption; AG, acylated ghrelin; TG, total ghrelin; PP, pancreatic polypeptide; PYY, peptide YY; GIP, glucose-dependent insulinotropic peptide; GLP-1, glucagon-like peptide 1; (i)AUC, (incremental) area under the curve; OGTT, oral glucose tolerance test; ↑, increase with exercise; ↓, decrease with exercise; →, no significant effect of exercise; (→), no significant effect of exercise at all other timepoints than those mentioned. ^1^ Exact measurement timepoint not mentioned. Note: If post hoc tests were not available, the authors estimated whether a significant change within a group was likely or not. If variances around the mean overlapped, no significant result was assumed.

**Table 6:** Effect of chronic exercise compared to control conditions (no training) on patients with type 2 diabetes mellitus (T2DM).

Study	Trial	Hunger	Fullness	PFC	Satiety	Spexin
Mohammadi et al. (2022) [52]	AT	→	→	→	→	↑ In the fasted state after 12 weeks
	RT	→	→	→	→	↑ In the fasted state after 12 weeks
Vidanage et al. (2023) [51] ^1^	AT	(→) ↓: 30 min pre-meal (in the fasted state) and 30 min post-meal after 6 months	-	-	(→) ↑: 30 min pre-meal (in the fasted state) after 6 months	-
	AT + RT	(→) ↓: 30 min pre-meal (in the fasted state) and 30 min post-meal after 6 months	-	-	(→) ↑: 30 min pre-meal (in the fasted state); 30 min and 60 min post-meal after 6 months	-

AT, aerobic endurance training; RT, resistance training; PFC, prospective food consumption; ↑, increase with exercise training; ↓, decrease with exercise training; →, no significant effect of exercise training; (→), no significant effect of exercise training at all other timepoints than those mentioned. ^1^ Results after 3 months are not shown in this table. Note: The study by Heiston et al. had no control group and was therefore not mentioned in this table.

## Data Availability

No new data were created or analyzed in this study. Data sharing is not applicable to this article. The data presented in this study are available in this article or via the references.

## Abbreviations

(d)AG	(Des-)acylated ghrelin
(i)AUC	(Incremental) area under the curve
AE	Aerobic endurance exercise
AT	Aerobic endurance training
BMI	Body mass index
CENTRAL	Cochrane Central Register of Controlled Trials
CINAHL	Cumulative Index to Nursing and Allied Health Literature
CKK	Cholecystokinin
CT.gov	ClinicalTrials.gov
GIP	Glucose-dependent insulinotropic peptide
GLP-1	Glucagon-like peptide-1
HbA1c	Glycated hemoglobin
HIIT	High-intensity interval training
HR_peak_	Maximal heart rate
ICTRP	International Clinical Trials Registry Platform
M	Meal
NGT	Normal glucose tolerance
OGTT	Oral glucose tolerance test
PEDro	Physiotherapy Evidence Database
PFC	Prospective food consumption
PICO	Patient Intervention Comparison Outcome
PP	Pancreatic polypeptide
PRISMA	Preferred Reporting Items for Systematic Reviews and Meta-Analyses
PYY	Peptide tyrosine tyrosine
RE	Resistance exercise
RM	Repetition maximum
RT	Resistance training
T2DM	Type 2 diabetes mellitus
TG	Total ghrelin
VO_2peak_	Maximal oxygen uptake
VT	Ventilatory threshold
W_max_	Maximal work capacity
